# Transcriptional responses of *Xanthomonas oryzae* pv. *oryzae* to type III secretion system inhibitor *ortho*-coumaric acid

**DOI:** 10.1186/s12866-019-1532-5

**Published:** 2019-07-15

**Authors:** Susu Fan, Fang Tian, Liwei Fang, Ching-Hong Yang, Chenyang He

**Affiliations:** 1 State Key Laboratory for Biology of Plant Diseases and Insect Pests Institute of Plant Protection, Chinese Academy of Agricultural Sciences, Beijing, 100193 China; 2 Department of Biological Sciences, University of Wisconsin-Milwaukee, Milwaukee, WI 53211 USA; 3 Shandong Provincial Key Laboratory of Applied Microbiology, Ecology Institute, Shandong Academy of Sciences, Jinan, 250014 Shandong Province China

**Keywords:** *Xanthomonas oryzae* pv. *oryzae*, Type III secretion system, Inhibitor, *ortho*-coumaric acid, Transcriptome

## Abstract

**Background:**

We previously identified a plant-derived phenolic compound *ortho*-coumaric acid (OCA) as an inhibitor of type III secretion system (T3SS) of *Xanthomonas oryzae* pv. *oryzae* (Xoo), the pathogen causing bacterial leaf blight of rice, one of the most devastating bacterial diseases of this staple crop worldwide. However, the molecular mechanisms by which OCA suppresses T3SS and the transcriptional responses to the OCA treatments in Xoo remains unclear.

**Results:**

The present study conducted the RNA-seq-based transcriptomic analysis to reveal changes in gene expression in Xoo in response to 30 min, 1 h, 3 h, and 6 h of OCA treatment. Results showed that OCA significantly inhibited the expression of T3SS genes after 30 min, and the inhibition also existed after 1 h, 3 h, and 6 h. After treatment for 30 min, membrane proteins in the functional category of cellular process was the predominant group affected, indicating that Xoo was in the early stress stage. Over time, more differentially-expressed genes (DEGs) gathered in the functional category of biological process. Analysis of common DEGs at all four of time points revealed the core elements of Xoo during the response to OCA treatment. Notable, a multidrug transporter cluster that consisted of a MarR-family protein (PXO_RS13760), a multidrug RND transporter (PXO_RS13755), a multidrug transporter (PXO_RS13750), and an MFS transporter (PXO_RS13745) were significantly up-regulated at all four of the time points. Although these three transporter genes were not upregulated by OCA in the PXO_RS13760 deletion mutant, the deficiency of PXO_RS13760 in Xoo did not affect T3SS transcript, and OCA still had the ability to inhibit the expression of T3SS in the mutant, suggesting that the MarR-family protein was involved in bacterial responses to OCA, but not direct OCA inhibition of T3SS in Xoo.

**Conclusions:**

We analyzed the transcriptome of Xoo during OCA treatment at both early and late stages, which revealed the landscape of Xoo responses to OCA at the whole-genome transcription level. A multidrug transporter cluster was identified to be involved in the response process, but had no direct relation to T3SS in Xoo.

**Electronic supplementary material:**

The online version of this article (10.1186/s12866-019-1532-5) contains supplementary material, which is available to authorized users.

## Background

Type III secretion system (T3SS) is an essential virulence mechanism in many gram-negative bacteria, and its structural components are highly conserved among different bacterial species [1–3]. Since anti-bacterial agents that target T3SS would affect pathogen virulence rather than viability, T3SS is also an attractive target for novel antimicrobials that generate low selective pressure for antimicrobial resistance development [4–6]. Furthermore, virulence factors are often absent in nonpathogenic bacteria, thereby limiting deleterious effects on endogenous microorganisms. Although several inhibitors of T3SS have been described in both animal and plant bacterial pathogens [4, 7–11], the mechanism of inhibition in each organism is not completely understood.

Salicylidene acylhydrazide (SAH) compounds are widely studied as T3SS inhibitors. In *Escherichia coli* O157:H7, a comprehensive analysis of transcriptional responses to four structurally related SAH compounds T3SS inhibitors were performed. The number of genes significantly affected markedly varied among different compounds [12], indicating that the inhibition mechanisms of different SAH compounds were not strictly the same [13]. Phenoxyacetamide was identified as a T3SS inhibitor of *Pseudomonas aeruginosa* [7]. PscF, which is the component of the needle apparatus, was proven to be the apparent molecular target of phenoxyacetamide MBX 1641 by isolating inhibitor-resistant mutants and mapping the mutation sites [14]. SAH compounds were also reported as T3SS inhibitors of plant pathogenic bacteria. Compounds 3 and 9 repressed the promoter activity of *hrpN*, *dspE*, *hrpL*, and *hrpA* in *Erwinia amylovora.* Compound 3 is capable of reducing disease development in crab apple flower [15].

Plant-derived phenolic compounds are another type of reported T3SS inhibitors of plant pathogenic bacteria. Umbelliferone (UM), a 7-hydroxycoumarin, suppressed T3SS regulator gene expression of *Ralstonia solanacearum* through the HrpG-HrpB and PrhG-HrpB pathways [16]. UM did not alter T3SS expression and swimming activity but significantly reduced biofilm formation. UM was observed to suppress the wilting disease process by reducing colonization and proliferation in tobacco roots and stems [16]. Recently, a set of plant extracts and molecules already described as T3SS inhibitors of bacterial pathogens of animals were found to suppress T3SS transcription in *R. solanacearum* [17].

*Ortho*-coumaric acid (OCA) and *trans*-cinnamic acid (TCA), which are also plant-derived phenolic compounds, were first reported as T3SS inducers of *Dickeya dadantii* [18]. An isomer of OCA, *p*-coumaric acid (PCA), was identified as T3SS inhibitors of *D. dadantii* [10]. A series of plant-derived phenolic compounds has been shown to regulate the expression of T3SS in *E. amylovora* and *P. aeruginosa* [9, 19]*.* In addition, 4-methoxy-cinnamic acid (TMCA) and benzoic acid (BA) suppressed hypersensitive responses of *E. amylovora* on non-host tobacco [9].

*Xanthomonas oryzae* pv. *oryzae* (Xoo), the causal agent of bacterial blight of rice, is one of the model systems that have been used to study the molecular mechanisms of bacterial pathogenesis in plants [20]. During the infection process, Xoo utilizes many different virulence factors to cause disease in susceptible hosts, including T3SS, extracellular polysaccharides (EPS), motility activity, and biofilm formation [21]. One of the main pathogenicity determinants in Xoo is T3SS, which secretes and delivers effector proteins (type III effectors, T3Es) into the plant cell. T3Es interact with molecules to manipulate plant cellular function, suppressing immunity and inducing the pathogen to multiply and spread across host plants. T3Es are also responsible for triggering the hypersensitive response (HR) in resistant or non-host plants [22, 23]. The T3SS of Xoo, which consists of more than 20 gene products, is tightly regulated. The expression of Xoo T3SS is induced *in planta* or in specially prepared minimal medium, which is designed to mimic *in planta* conditions, while it is suppressed in nutrient-rich medium [24].

We previously reported that OCA inhibits the T3SS of Xoo, neutralizing the virulence but not affecting the growth of the organism [25]. OCA is the precursor compound of salicylic acid (SA), which plays an important role in plant defense responses [26]. The water soaking symptoms and disease symptoms caused by Xoo strain PXO99^A^ were reduced after treatment by OCA. However, the components and pathways that mediate the effect of OCA on T3SS remains unclear. In this study, we performed transcriptomic analysis to reveal gene expression changes of Xoo in response to the treatment of OCA at both early and late stages, which revealed the landscape of Xoo response to OCA at the whole-genome transcription level. In addition, a MarR-family protein was identified to be involved in the response process, but had no direct relation to T3SS in Xoo.

## Results

### OCA inhibited the expression of T3SS even after it is already induced

It has previously been shown that the expression of T3SS-related genes in Xoo is significantly inhibited by OCA [25]. As a primary virulence factor, T3SS is induced at the early stage of infection. Therefore, we first investigated whether OCA could suppress T3SS expression even after it is induced. We first induced the T3SS gene expression in Xoo using minimal medium XOM2 for 1 h to mimic the natural infection process. Six representative T3SS related genes were selected to show the results by quantitative real-time PCR (qRT-PCR). The expression of the five genes was notably induced after inoculating in XOM2 for 1 h except for *hrcC*, which was not induced until after 6 h (Additional file 1: Figure S1). After 1 h induction, OCA was added to the medium at a concentration of 200 μM, and equal volume DMSO was used as solvent control. The inhibition of *hpa1*, *hrpF* and *hrcT* was observed 2 h after OCA treatment, *hrpX* was after 4 h, while *hrpG* and *hrcC* was observed after 6 h, respectively (Additional file 1: Figure S1). Based on the results, we speculated that the response of Xoo to OCA was accomplished within 6 h.

### RNA-seq analysis of Xoo under the treatment of OCA

**Fig. 1:**
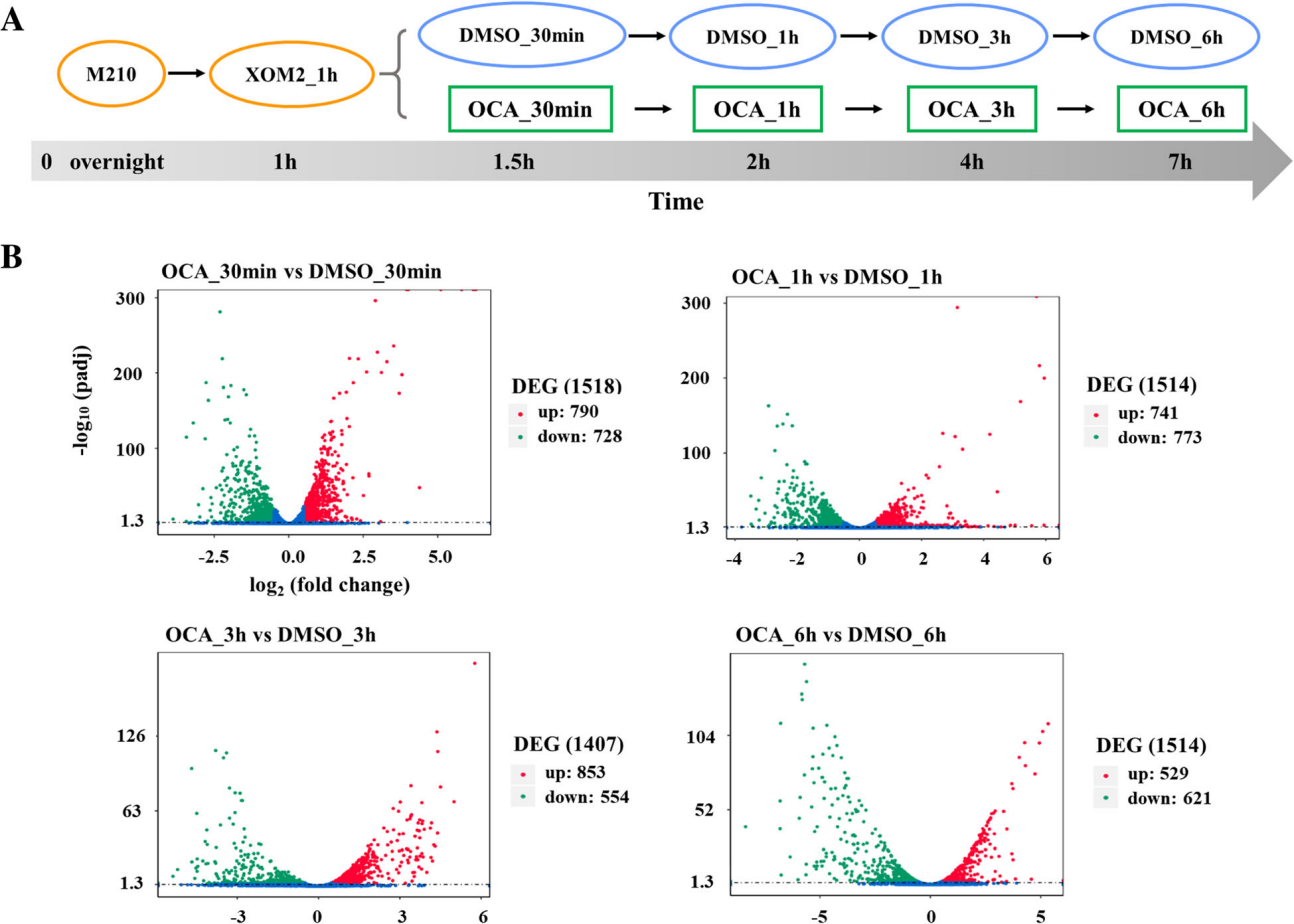
Establishment of transcriptome analysis under OCA treatment. **a** Workflow of transcriptome analysis. Xoo strain PXO99^A^ was incubated in M210 medium overnight, then resuspended in XOM2 medium at an OD_600_ of 0.6. 200 μM OCA or equal volume DMSO was added to the culture after 1 h, and total mRNA was extracted at 30 min, 1 h, 3 h and 6 h after OCA or DMSO treatment, respectively. **b** Number of DEGs at each time point was presented by scatter diagram

**Fig. 2:**
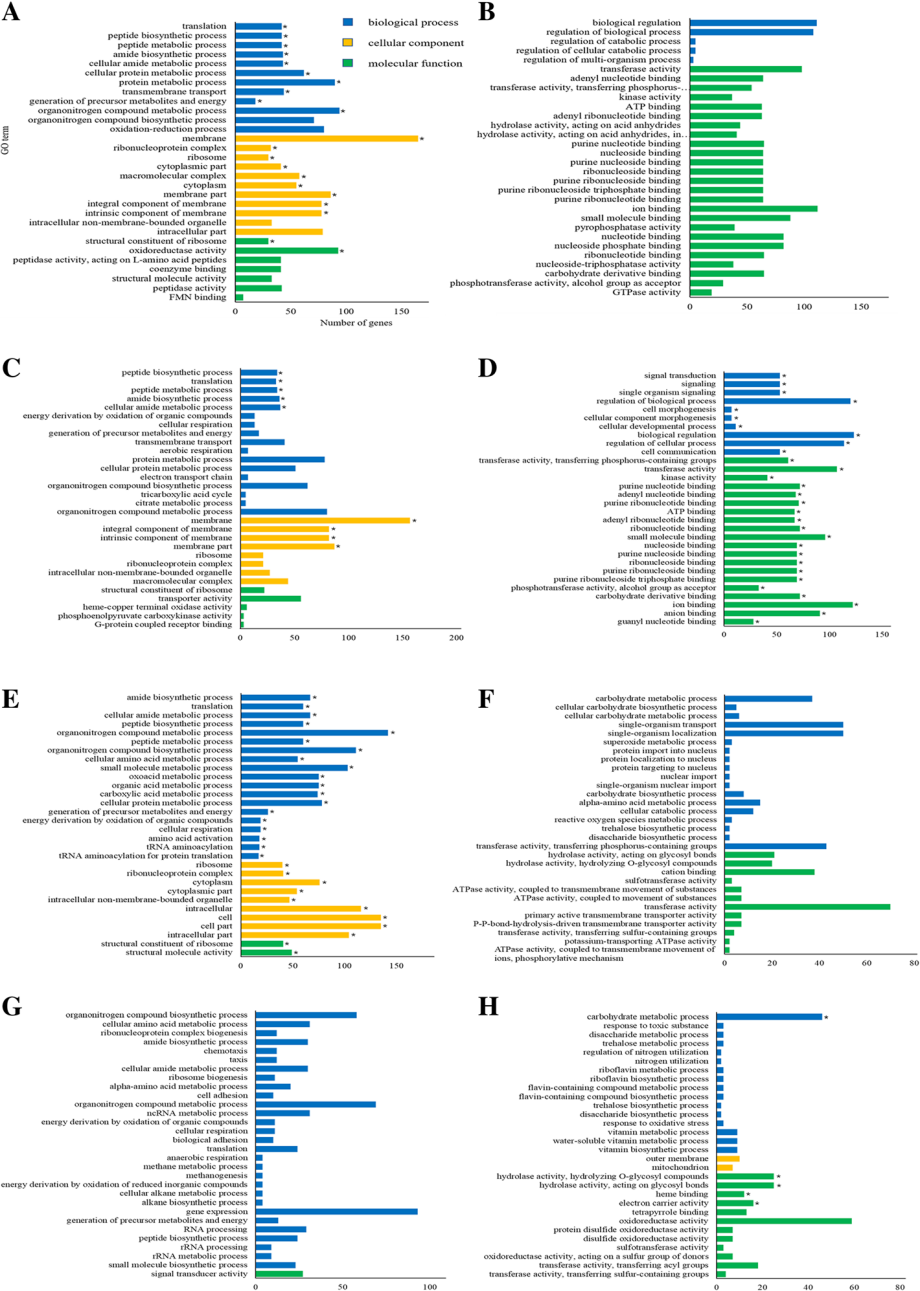
GO analyses of up-regulated and down-regulated genes at each time point. **a** Go analyses of up-regulated genes at 30 min after OCA treatment; **b** Go analyses of down-regulated genes at 30 min after OCA treatment; **c** Go analyses of up-regulated genes at 1 hour after OCA treatment; **d** Go analyses of down-regulated genes at 1 hour after OCA treatment; **e** Go analyses of up-regulated genes at 3 h after OCA treatment; **f** Go analyses of down-regulated genes at 3 h after OCA treatment; **g** Go analyses of up-regulated genes at 6 h after OCA treatment; **h** Go analyses of down-regulated genes at 6 h after OCA treatment

To investigate transcriptional responses of Xoo to OCA, transcriptomic analyses were conducted on RNAs extracted from Xoo after treatment with OCA for 30 min, 1 h, 3 h, and 6 h, respectively (Fig. 1a). DEGs at each time point were identified according to the standards below: absolute value of log_2_FC > 0.5 and *p*-value (adjusted) < 0.05. The results showed that OCA had remarkably influenced the transcriptome of Xoo. Approximately 30 min after treatment, 1518 DEGs were identified relative to the DMSO control, and the number of DEGs was 1514, 1407, and 1514 at 1 h, 3 h, and 6 h after treatment, respectively (Fig. 1b and Additional file 2: Figure S2). About 30% of the total number of genes of the PXO99^A^ genome (5058 genes) showed differential expression at all four time points. Using Gene Ontology (GO) DEG classifications, the DEGs were categorized into three functional groups, including biological process, cellular component, and molecular function. After 30 min of OCA treatment, the majority of the up-regulated genes were functionally associated with the membrane, and downregulated genes were highly enriched in the molecular function term of the GO database (Fig. 2a and b). The DEGs at 1 hour after treatment showed similar enrichment as that of 30 min, and more genes in biological process term of GO database were upregulated than that of 30 min (Fig. 2c and d). After treatment with OCA for 3 h, many more genes in biological process were affected; meanwhile, genes associated with membrane were not significantly upregulated any more (Fig. 2e and f). In the case of 6 h of treatment, almost all of the upregulated genes were gathered in the biological process term, while most of the downregulated genes were gathered in both biological process and molecular function terms (Fig. 2g and h).

### T3SS-related genes are down-regulated under OCA treatment at all four of time points

**Fig. 3:**
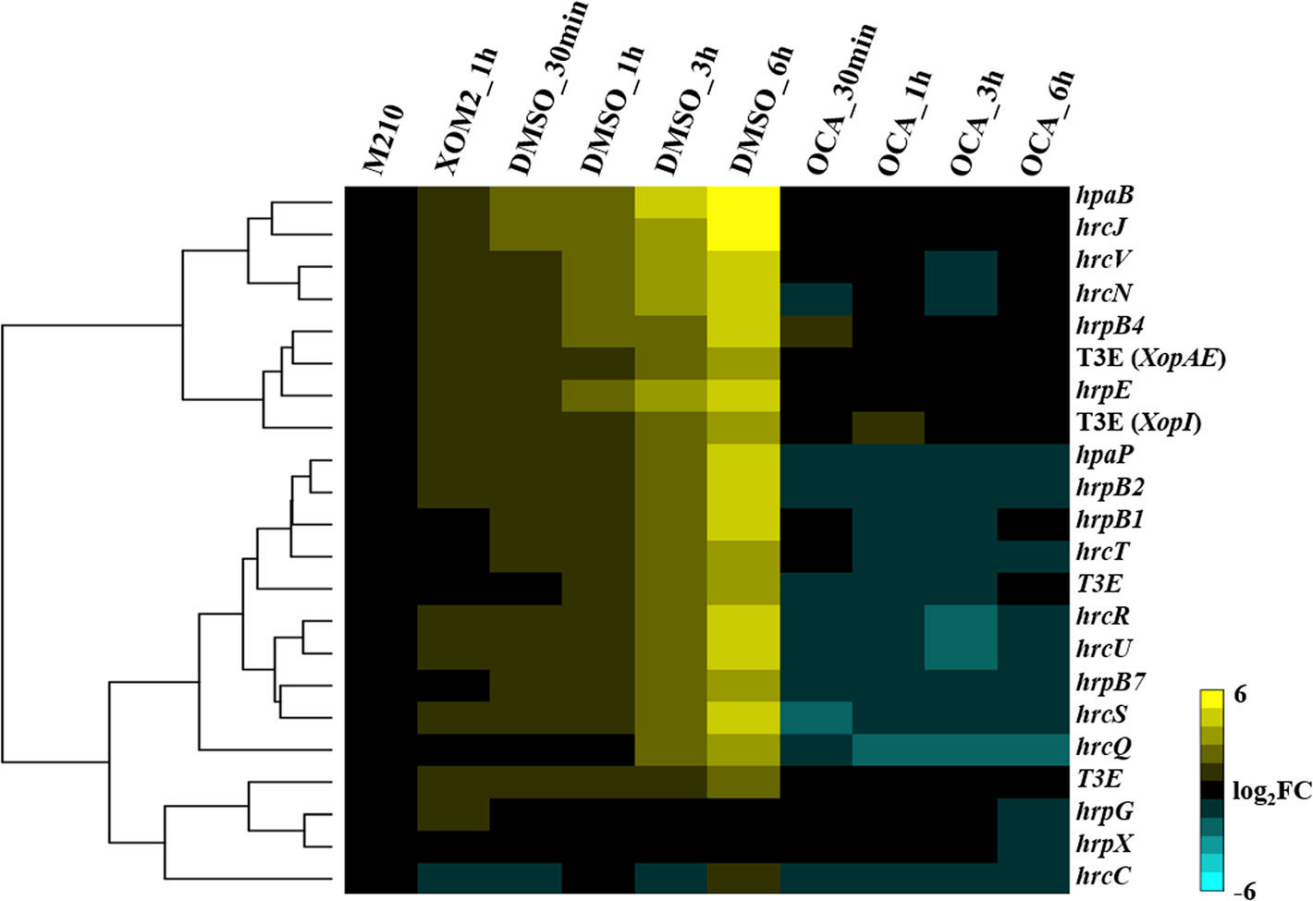
Heat map of T3SS associated genes expression. Compared with M210 medium, most of T3SS associated genes expression were induced by XOM2 medium after one-hour treatment, the level of their expression risen continuously as time extended. OCA strongly inhibited the expression of these genes from 30 min after treatment, which was the first time point tested, and the effect of OCA existed until 6 hours after treatment, which was the last time point tested

Since we expected the expression of T3SS-related genes should be downregulated, we specially assessed their expression patterns in the RNA-seq data. A total of 22 genes from the *hrp*/*hrc*/*hpa* gene cluster, the regulatory genes *hrpG* and *hrpX*, as well as four T3Es were inspected. First, we found that relative to M210 treatment, most of the T3SS genes were induced after grown in XOM2 for 1 h (Fig. 3). Compared with the DMSO control, OCA significantly inhibited the expression of these genes at 30 min after treatment, and this continued until 6 h, which was consistent with the results of qRT-PCR (Fig. 3 and Additional file 1: Figure S1).

### OCA does not inhibit the expression of flagellar genes

**Fig. 4:**
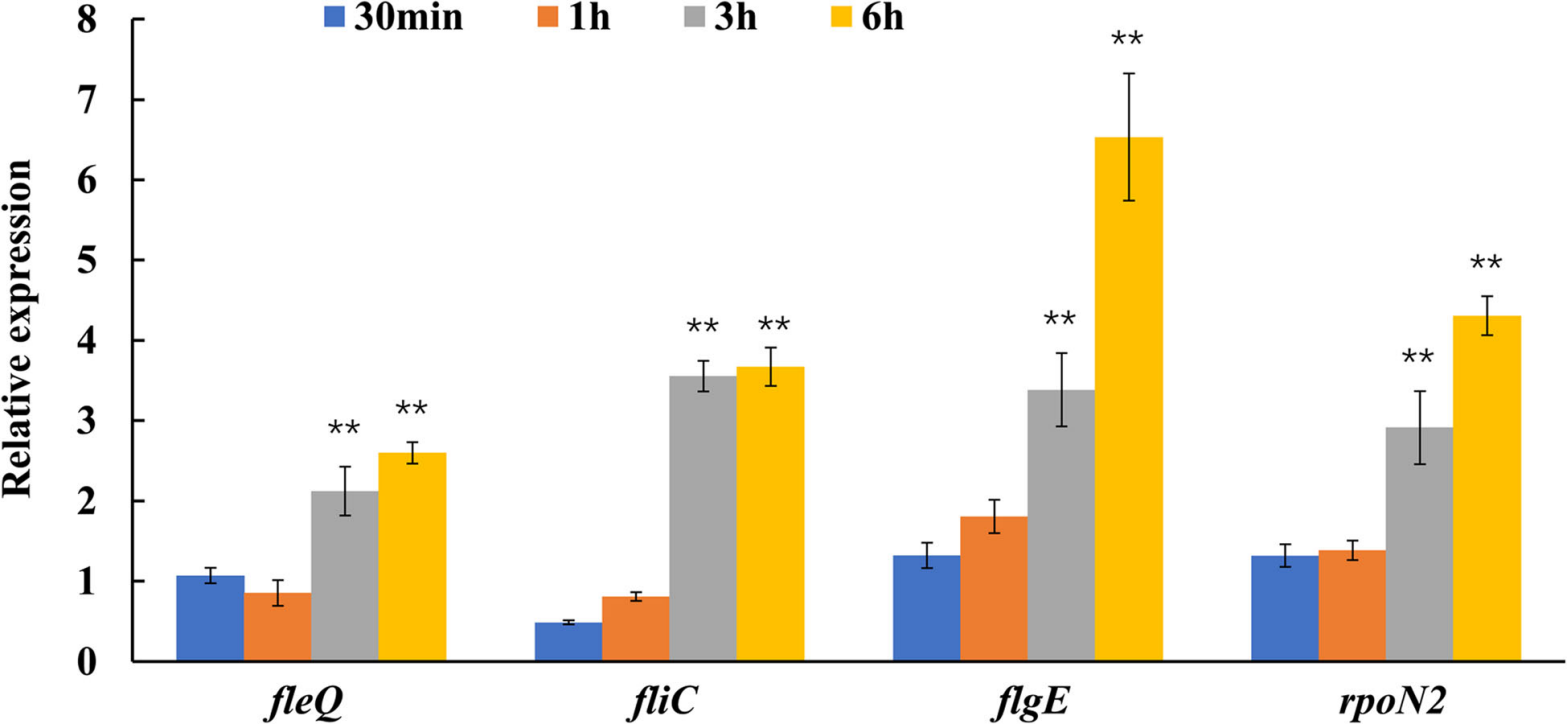
Relative mRNA levels of four representative genes from flagella cluster of Xoo under OCA treatment were measured by qRT-PCR. OCA had no significant effect on the expression of these four genes within 1 hour. The expression of them was induced by OCA after 3 hours treatment, and the influence was more obviously after 6 hours. Values represent the levels of expression compared to that of bacterial cells without OCA treatment (for which the level of expression was set equal to 1.00). Asterisks indicate statistically significant differences in the expression levels between bacterial cells treated with or without OCA (Student’s t-test). ** *P* < 0.01

As T3SS and the flagellar system are highly similar in terms of their structure and gene homology, we investigated whether OCA also affected the expression of flagellar genes. In Xoo PXO99^A^, the flagellar gene cluster is located within a region that consists of two copies in the genome. Due to the algorithm that we used for RNA-seq data analysis discarding sequences corresponding to more than one genomic locus, flagellar genes were not included in the final results. To find out how flagellar genes respond to OCA treatment, we performed qRT-PCR analysis on four representative flagellar genes, which included the sigma-54 factor gene *rpoN2*, sigma-54-dependent transcriptional regulator gene *fleQ*, the flagellin gene *fliC*, and the flagellar hook gene *flgE*. The same batch of cDNA, which were used for validating the DEGs, were used to test the expression of flagellar genes at all four time points after treatment by OCA. The results showed that the expression of four flagellar genes did not significantly change within an hour of OCA treatment. Interestingly, these genes were induced by OCA after 3 h (Fig. 4).

### Analysis of common DEGs at all four of time points revealed the core elements of Xoo during the response to OCA treatment

**Fig. 5:**
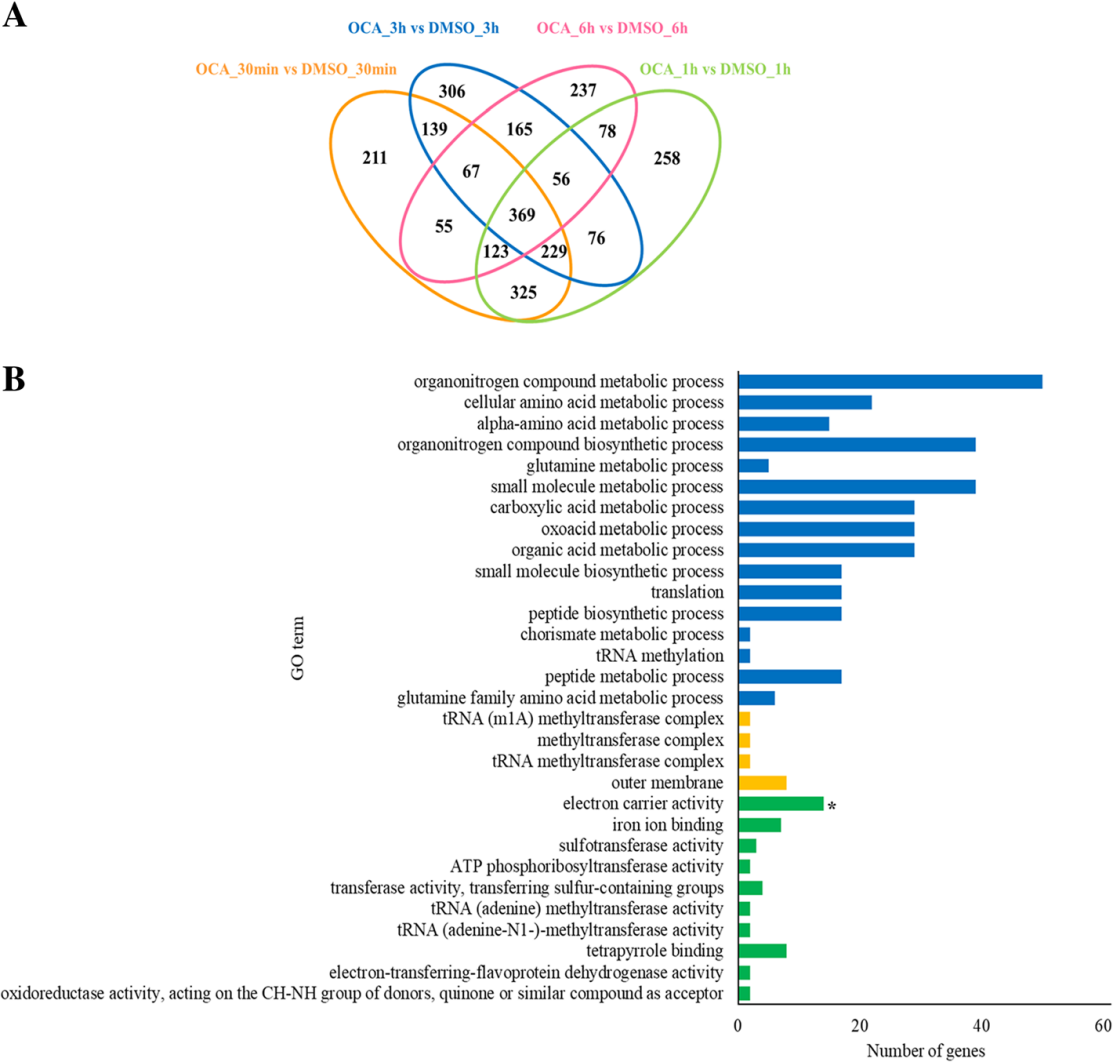
At all four of time points, 369 genes were common regulated by OCA. **a** Number of DEGs at each time point was presented by Venn diagram; **b** Go analysis of 369 common regulated genes. Genes associated with electron carrier activity was significant enrichment

To reveal the core elements responding to OCA treatment, 369 common DEGs at all four of time points were identified (Fig. 5a). Among these, 170 DEGs were upregulated and 199 DEGs were downregulated (Additional file 3: Table S1). To validate RNA-seq data, qRT-PCR was performed with 32 arbitrarily selected up- or down-regulated genes among the 369 common DEGs of four time points. A significant correlation was observed between the relative expression levels determined by RNA-seq and qRT-PCR (Additional file 4: Figure S3).

Base on GO analysis, genes associated with electron carrier activity in molecular function were notably gathered (Fig. 5b, and Additional file 5: Table S2). Since GO analysis was not able to classify these DEGs into different functional groups, we assessed the annotation of each gene. We found over 100 genes were functionally associated with pathogenic processes, including secretion systems, oxidation-reduction reactions, transporters, membrane proteins, transcriptional regulators, signaling pathways, cytochrome, and chemotaxis (Table 1). Among them, 24 genes apparently formed five gene clusters (Table 2).

**Table 1:** Common DEGs associated with bacterial pathogenesis processes

Classifications	Gene Numbers
Secretion systems	27
Oxidation-reduction Reactions	22
Transporters	16
Membrane proteins	12
Transcriptional regulators	12
Signaling pathways	9
Cytochrome	6
Chemotaxis	4

**Table 2:** Common differential expression gene clusters

Cluster	Gene ID	Gene Description
I	PXO_RS07140	ubiquinol oxidase subunit II
	PXO_RS07145	cytochrome o ubiquinol oxidase subunit I
	PXO_RS07150	cytochrome o ubiquinol oxidase subunit III
	PXO_RS07155	cytochrome o ubiquinol oxidase subunit IV
II	PXO_RS08700	TetR/AcrR family transcriptional regulator
	PXO_RS08695	efflux RND transporter periplasmic adaptor subunit
	PXO_RS08690	multidrug efflux RND transporter permease subunit
III	PXO_RS13760	MarR family transcriptional regulator
	PXO_RS13755	multidrug RND transporter
	PXO_RS13750	multidrug transporter
	PXO_RS13745	MFS transporter
IV	PXO_RS16460	NADH-quinone oxidoreductase subunit B
	PXO_RS16455	NADH-quinone oxidoreductase subunit C
	PXO_RS16450	NADH-quinone oxidoreductase subunit D
	PXO_RS16445	NADH-quinone oxidoreductase subunit NuoE
	PXO_RS16440	NADH-quinone oxidoreductase subunit F
	PXO_RS16435	NADH dehydrogenase (quinone) subunit G
	PXO_RS16430	NADH-quinone oxidoreductase subunit H
	PXO_RS16425	NADH-quinone oxidoreductase subunit I
V	PXO_RS10645	succinate dehydrogenase, cytochrome b556 subunit
	PXO_RS10650	succinate dehydrogenase, hydrophobic membrane anchor protein
	PXO_RS10655	succinate dehydrogenase flavoprotein subunit
	PXO_RS10665	succinate dehydrogenase iron-sulfur subunit
	PXO_RS10670	succinate dehydrogenase assembly factor 2 family protein

### PXO_RS13760 is involved in the response of Xoo to OCA

**Fig. 6:**
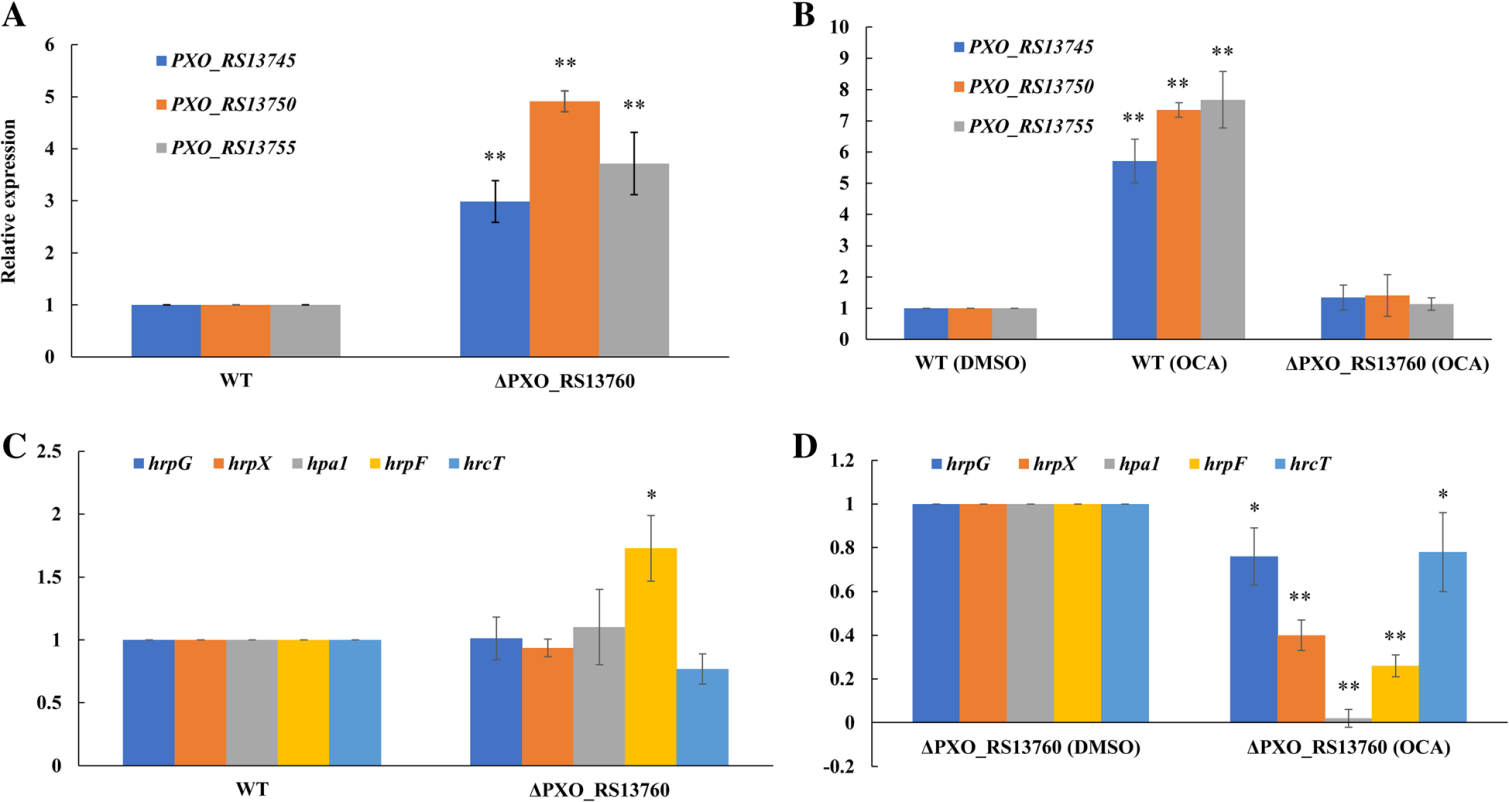
PXO_RS13760 was involved in the response of OCA in Xoo but had no relationship with the regulation of T3SS. **a** Relative mRNA levels of *PXO_RS13745*, *PXO_RS13750* and *PXO_RS13755* in wild type and *PXO_RS13760* deletion mutant were measured by qRT-PCR. Xoo cells were cultured in M210 medium overnight and subcultures to XOM2 medium for 6 hours. Values represent the levels of expression compared to that of wildtype (for which the level of expression was set equal to 1.00); **b** Relative mRNA levels of *PXO_RS13745*, *PXO_RS13750* and *PXO_RS13755* in wild type and *PXO_RS13760* deletion mutant under OCA treatment were measured by qRT-PCR. Xoo cells were cultured in M210 medium overnight and subcultures to XOM2 medium. After 1 h incubation, OCA was added to the medium at a concentration of 200 μM and incubated for 6 h. Values represent the levels of expression compared to that of wildtype without OCA treatment (for which the level of expression was set equal to 1.00); **c** Relative mRNA levels of *hrp* genes in wildtype and *PXO_RS13760* deletion mutant were measured by qRT-PCR. Xoo cells were cultured in M210 medium overnight and subcultures to XOM2 medium for 6 hours. Values represent the levels of expression compared to that of wildtype (for which the level of expression was set equal to 1.00); **d** Relative mRNA levels of *hrp* genes in *PXO_RS13760* deletion mutant under OCA treatment were measured by qRT-PCR. Xoo cells were cultured in M210 medium overnight and subcultures to XOM2 medium. After 1 h incubation, OCA was added to the medium at a concentration of 200 μM and incubated for 6 h. Values represent the levels of expression compared to that of bacterial cells without OCA treatment (for which the level of expression was set equal to 1.00). Asterisks indicate statistically significant differences in the expression levels (Student’s t-test). * *P* < 0.05; ** *P* < 0.01

A multidrug transporter cluster which composed of a MarR-family protein (PXO_RS13760), a multidrug RND transporter (PXO_RS13755), a multidrug transporter (PXO_RS13750), and an MFS transporter (PXO_RS13745) was significantly upregulated at all four time points (Table 3). According to previous reports [27, 28], MarR-family proteins are always involved in binding to phenolic compounds and pathogenic process regulations. As a result, we constructed an in-frame deletion mutant of the above-mentioned MarR-family protein. Transcripts for these three transporter proteins were up-regulated in the mutant (Fig. 6a), and their expression were not induced by OCA in the mutant, indicating this MarR-family protein has an important role in Xoo during the response to OCA (Fig. 6b). However, the deficiency of this protein in Xoo did not significantly affect *hrp* gene expression (Fig. 6c), and OCA still had the ability to inhibit the expression of T3SS in the mutant (Fig. 6d), suggesting this MarR-family protein might not be involved in the regulation of T3SS in Xoo.

**Table 3:** A multidrug transporter cluster up-regulated at all four of time points

Gene ID	Log_2_ Fold Change				Gene Description
	30 min	1 h	3 h	6 h	
PXO_RS13745	3.7987	4.1977	3.8392	4.9377	MFS transporter
PXO_RS13750	5.0991	5.1852	3.8392	5.3337	Multidrug transporter
PXO_RS13755	5.7991	5.9487	3.3722	5.0849	Multidrug RND transporter
PXO_RS13760	4.386	5.9487	2.8828	3.4645	MarR family transcriptional regulator

## Discussion

Phenolic compounds are one of the major classes of secondary metabolites found in the plant. About 10,000 structures of phenolics have been identified to date [29]. These compounds are usually involved in inducing plant resistance to prevent pathogenic bacteria, as well as in the interaction between pathogen and host. Our previous studies indicated that a small phenolic compound OCA, a precursor of SA [26], was able to suppress expression of the T3SS-associated genes in Xoo [25]. The function of T3SS in Xoo was almost completely suppressed by OCA treatment, thus leading to attenuated virulence on rice. In this study, we performed RNA-seq analysis of Xoo in the presence of OCA and compared the gene expression patterns at different stages of OCA treatment. According to the results of our pre-experiment (Additional file 1: Figure S1) and the previous report, expression of T3SS genes can be induced within 1 hour after incubation in a T3SS-inducing medium or rice leaf extract [30]. The results presented here indicated that OCA can neutralize the influence of T3SS-inducing medium, which makes OCA more suitable for practical application in the environment.

In agreement with the previous studies examining repression of T3SS, the inhibition of OCA on T3SS gene expression could be observed at 30 min after treatment, which was the first time point that was tested (Fig. 3). This result suggests that the response of Xoo for OCA occurred over a short time period. The majority of the DEGs at 30 min after treatment could be classified to membrane protein, which might explain this rapid response (Fig. 2). The affinity of OCA on T3SS could still be observed at 6 h after treatment, even though the DEGs detected at this time point showed no significant enrichment in the membrane protein functional category (Fig. 2). However, more genes associated with biological process showed differential expression, suggesting OCA has a deeper impact on the intracellular physiological activities of Xoo (Fig. 2).

A total of 369 genes were differentially expressed at all four time points tested (Fig. 5). Among them, a multidrug transporter cluster was markedly upregulated by OCA treatment (Table 3). As the other three T3SS inhibitors reported previously showed similar influence on the expression of this cluster (Additional file 6: Figure S4), it was supposed to be the target of OCA in Xoo to inhibit the *hrp* gene expression. Not as expected, the deletion of *PXO_RS13760* showed no significant influence on the expression of *hrp* genes (Fig. 6c and d). Although the MarR family proteins had also been reported to be involved in multiple antibiotic resistance phenotypes and the control of multidrug efflux pumps [31], the absence of PXO_RS13760 in Xoo also did not lead it to be more sensitive to OCA (Additional file 7: Figure S5).

Two-component regulatory systems (TCSs), which are composed of a membrane-bound histidine kinase that senses a specific environmental stimulus and a corresponding response regulator that mediates the cellular response, serve as a basic stimulus-response mechanism to cope with various environmental conditions [32]. In this study, two transcripts (*PXO_RS07440* and *PXO_RS08715*) associated with TCSs were downregulated at all four time points, whose role in the regulation of T3SS in Xoo has not been investigated (Additional file 8: Table S3). Several proteins were reported to influence the expression of *hrp* genes in Xoo, such as Rpfc/RpfG, Pdek/PdeR, GdpX1, PXO_00049, PXO_02374 and Filp [33–37], but none of them showed obviously differential expression under OCA treatment, indicating there are other regulatory pathways of T3SS in Xoo.

Iron uptake is vital for bacterial survival and cellular metabolism [38]. Siderophores are transported back into bacterial cells through an outer membrane receptor, which is regulated by other membrane-associated proteins, such as TonB [39, 40]. Our results also showed that several iron-uptake associate genes were differentially expression under OCA treatment (Additional file 9: Table S4).

As one of the most widely studied T3SS inhibitors, the global transcriptional response of pathogenic bacteria to SAH were also analyzed. INP0403, a member of SAH compounds, had been identified as a T3SS inhibitor of *Salmonella enterica* serovar Typhimurium. In addition to genes involved in T3SS, INP0403 treatment also affected the transcription of genes associated with iron acquisition [41]. The SAH T3SS inhibitors of *Chlamydia* have also been reported to be directly or indirectly linked with iron [42]. To elucidate the molecular mechanism by which SAH class compounds affect the T3SS, 16 putative target proteins of ME0052 and ME0055, which are SAH family T3SS inhibitors of *E. coli* O157:H7, were identified by affinity chromatography [43]. WrbA, Tpx, and FolX, as well as their homologous proteins from other bacterial species, were verified to have direct interactions with ME0052 and ME0055 by far-western blotting. These three proteins were involved in the conservative metabolic pathways rather than T3SS, but the deletion mutants of the corresponding gene encoding each protein in both *E. coli* O157 and *Yersinia pseudotuberculosis* stimulated the expression of T3SS. In *Erwinia amylovora*, the majority of T3SS genes were suppressed under treatment of compounds 3 and 9. However, the expression of amylovoran biosynthesis genes also decreased [15]. These results suggest that SAH compounds repress T3SS through their influence on global biological processes, rather than directly affect T3SS expression. Our data revealed that the effect of OCA in Xoo was also global instead of specifically towards a few genes. It should be noted that although the in vitro system allowed us to control the interactions between plant and pathogen in a time-dependent manner, to some extent, the interactions in the in vitro system could differ from the real in vivo interactions.

## Conclusion

We demonstrated the transcriptomic responses of Xoo under OCA treatment at both the early and late stage for the first time, which revealed the landscape of Xoo responses to OCA at the whole-genome transcription level. Analysis of common DEGs at all four of time points revealed the core elements of Xoo during the response to OCA treatment. In addition, a MarR-family protein was identified to be involved in the response process but had no direct relation to T3SS in Xoo.

## Materials and methods

### Bacterial strains and culture conditions

Xoo wild-type strain PXO99^A^ (lab collection) and the derived strains were grown in M210 medium (0.8% casein enzymatic hydrolysate, 0.5% sucrose, 0.4% yeast extract, 17.2 mM K_2_HPO_4_, 1.2 mM MgSO_4_·7H_2_O) or on PSA plates. XOM2 medium [0.18% D-(+) xylose, 670 μM L-methionine, 10 mM sodium L-(+) glutamate, 14.7 mM KH_2_PO_4_, 40 μM MnSO_4_, 240 μM Fe(III)-EDTA, and 5 mM MgCl_2_, the pH was adjusted to 6.5 with KOH] was used for *hrp*-inducing conditions [44].

### Construction of gene deletion mutant

An in-frame deletion mutation of the *PXO_RS13760* gene was constructed in PXO99^A^ through homologous recombination using the suicide vector pKMS1, as described previously [45]. The *sacB* gene (sucrose sensitivity counter-selectable marker) on pKMS1 confers suicide ability to the host bacterium during growth on high-concentration sucrose-containing medium. Briefly, approximately 600 base pairs of the upstream and 900 base pairs of the downstream region of the *PXO_RS13760* gene were amplified from PXO99^A^ genomic DNA using the primer pairs USF/R and DSF/R, respectively. The primers used in this study are listed in Table S5 (Additional file 10: Table S5). The two fragments were ligated into the suicide vector pKMS1 and introduced into PXO99^A^ by electroporation. The transformants were first selected on NAN medium (consisting of kanamycin, 1% tryptone, 0.1% yeast extract, 0.3% peptone, 1.5% agar). After continuous transfer culture in NBN broth (1% tryptone, 0.1% yeast extract, 0.3% peptone) for four times, the mutant candidates that grew on NAS, but were sensitive to kanamycin, were further confirmed by PCR.

### RNA extraction and qRT-PCR analysis

Xoo cells were cultured in M210 medium overnight at 28 °C and subcultures to XOM2 medium at an optical density at 600 nm (OD_600_) of 0.3. After 1 h incubation, each compound needed was added to the medium at a concentration of 200 μM, and equal volume DMSO was used as solvent control. Total RNA was isolate using an RNAprep Pure Bacteria Kit (Tiangen, Beijing, China). cDNA was synthesized using an HiScriptll Q RT SuperMix Kit (Vazyme, Nanjing, China). The cDNA levels of different samples were quantified by qRT-PCR using a SYBR Green Master Mix (Vazyme, Nanjing, China). The relative levels of gene expression were determined using the 2^-ΔΔCT^ method [46], with the DNA gyrase subunit B (*gyrB*) gene as the internal control [38]. Three technical replicates were used each time.

### RNA sequencing and data analysis

A total amount of three μg RNA per sample was used as input material for the RNA sample preparations. Sequencing libraries were generated using NEBNext® Ultra™ Directional RNA Library Prep Kit for Illumina® (NEB, USA) following manufacturer’s recommendations and index codes were added to attribute sequences to each sample. The clustering of the index-coded samples was performed on a cBot Cluster Generation System using TruSeq PE Cluster Kit v3-cBot-HS (Illumina) according to the manufacturer’s instructions. After cluster generation, the library preparations were sequenced on an Illumina HiSeq platform and paired-end reads were generated. The RNA sequencing was performed by Novogene (Beijing, China). Raw data (raw reads) in the fastq format were first processed through in-house perl scripts. In this step, clean data (clean reads) were obtained by removing reads containing adapter, reads containing ploy-N and low-quality reads from raw data. Reference genome and gene model annotation files were downloaded from genome website directly (https://www.ncbi.nlm.nih.gov/nuccore/NC_010717.2). Both building index of reference genome and aligning clean reads to reference genome used Bowtie2–2.2.3 [47]. HTSeq v0.6.1 was used to count the reads numbers mapped to each gene. Then FPKM of each gene was calculated based on the length of the gene and reads count mapped to this gene. The RNA-Seq data has been deposited in NCBI Sequence Read Archive (SRA) with the SRA Series accession number PRJNA505936 (https://www.ncbi.nlm.nih.gov/sra/PRJNA505936). Differential expression analysis of two groups was performed using the DESeq R package (1.18.0). The resulting *P*-values were adjusted using the Benjamini and Hochberg’s approach for controlling the false discovery rate. Genes with an adjusted P-value < 0.05 found by DESeq were assigned as differentially expressed. GO enrichment analysis of differentially expressed genes was implemented by the GOseq R package, in which gene length bias was corrected. GO terms with corrected P-value less than 0.05 were considered significantly enriched by differential expressed genes. The analysis of sequencing data, including identification of DEGs and Go enrichment analysis were performed using NovoMagic platform. The heatmap was generated using the ‘gplots’ package in R with log_2_FC values [48].

## Additional files

Additional file 1: **Figure S1.** Relative mRNA levels of *hrp* genes in Xoo PXO99^A^ incubated with OCA were measured by qRT-PCR. Xoo cells were cultured in M210 medium overnight and subcultures to XOM2. After 1 h incubation, OCA was added to the medium at a concentration of 200 μM, and equal volume DMSO was used as solvent control. A: M210 vs XOM2_1 h; B: DMSO_2h vs OCA_2h; C: DMSO_4h vs OCA_4h; D: DMSO_6 h vs OCA_6 h; E: DMSO_8h vs OCA_8h. Values represent the levels of expression compared to that of bacterial cells cultured in M210 medium overnight (for which the level of expression was set equal to 1.00). Asterisks indicate statistically significant differences in the expression levels (Student’s t-test). * *P* < 0.05; ** *P* < 0.01. (TIF 10006 kb)

Additional file 2: **Figure S2.** Heatmap of OCA effects on Xoo PXO99^A^ transcriptome. (TIF 10232 kb)

Additional file 3: **Table S1.** Common DEGs at all four of time points. (XLSX 47 kb)

Additional file 4: **Figure S3.** Correlation analysis between qRT-PCR and RNA-seq data of 32 randomly selected genes. A, B, C and D represented 30 min, 1 h, 3 h and 6 h after OCA treatment, respectively. (TIF 215 kb)

Additional file 5: **Table S2.** Common DEGs at all four of time points associated with electron carrier activity. (XLSX 9 kb)

Additional file 6: **Figure S4.** Relative mRNA levels of the multidrug transporter gene cluster under treatment of different T3SS inhibitors were measured by qRT-PCR. (A) Relative mRNA levels of the multidrug transporter gene cluster in Xoo PXO99^A^ incubated with four T3SS inhibitors respectively. (B) Relative mRNA levels of the multidrug transporter gene cluster in Δ*PXO_RS13760* incubated with four T3SS inhibitors respectively. (TIF 10256 kb)

Additional file 7: **Figure S5.** Relative mRNA levels of *hrp* genes in Xoo incubated with OCA under different concentrations were measured by qRT-PCR. WT0, Xoo PXO99^A^ wild type strain incubated without OCA; WT10, Xoo PXO99^A^ wild type strain incubated with 10 μM OCA; WT50, Xoo PXO99^A^ wild type strain incubated with 50 μM OCA; WT100, Xoo PXO99^A^ wild type strain incubated with 100 μM OCA; M0, *PXO_RS13760* deletion mutant incubated without OCA; M10, *PXO_RS13760* deletion mutant incubated with 10 μM OCA; M50, P*XO_RS13760* deletion mutant incubated with 50 μM OCA; M100, *PXO_RS13760* deletion mutant incubated with 100 μM OCA. (TIF 9587 kb)

Additional file 8: **Table S3.** Signaling pathway and transcriptional regulators which were common regulated at all four of time points by OCA. (XLSX 11 kb)

Additional file 9: **Table S4.** Iron metabolism related genes which were regulated by OCA at each time point. (XLSX 12 kb)

Additional file 10: **Table S5.** Primers used in this study. (DOCX 25 kb)

## Data Availability

The RNA-Seq data has been deposited in NCBI Sequence Read Archive (SRA) with the SRA Series accession number PRJNA505936 (https://www.ncbi.nlm.nih.gov/sra/PRJNA505936). Other data generated or analyzed during this study are included in this published article and its additional files.

## Abbreviations

BA: Benzoic acid
DEGs: Differentially expressed genes
EPS: Extracellular polysaccharides
Go: Gene Ontology
*gyrB*: Gyrase subunit B
HR: Hypersensitive response
OCA: *Ortho*-coumaric acid
OD_600_: Optical density at 600 nm
PCA: *P*-coumaric acid
qRT-PCR: Quantitative real-time PCR
SA: Salicylic acid
*sacB*: Sucrose sensitivity counter-selectable marker
SAH: Salicylidene acylhydrazide
SRA: Sequence Read Archive
T3Es: Type III effectors
T3SS: Type III secretion system
TCA: *Trans*-cinnamic acid
TCSs: Two-component regulatory systems
TMCA: 4-methoxy-cinnamic acid
UM: Umbelliferone
Xoo: *Xanthomonas oryzae* pv. *oryzae*
