# Supplementary figures and images for: Transcriptional responses of Xanthomonas oryzae pv. oryzae to type III secretion system inhibitor ortho-coumaric acid
Source: BMC Microbiol. 2019 Jul 15;19:163. doi: 10.1186/s12866-019-1532-5 (PMC6631524; doi:10.1186/s12866-019-1532-5)

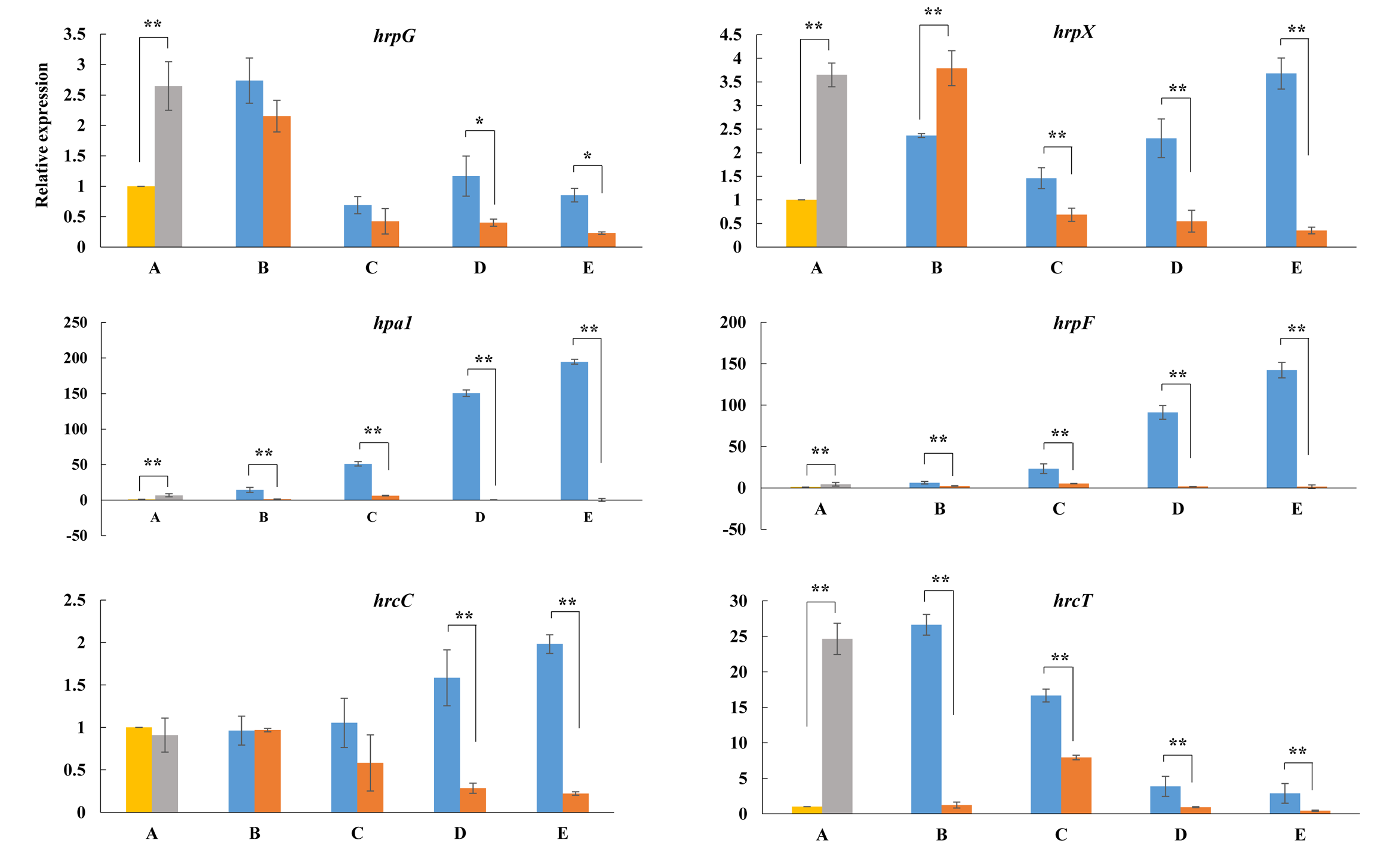

Supplement: Supplementary file 1 — Figure S1. Relative mRNA levels of hrp genes in Xoo PXO99A incubated with OCA were measured by qRT-PCR. Xoo cells were cultured in M210 medium overnight and subcultures to XOM2. After 1 h incubation, OCA was added to the medium at a concentration of 200 μM, and equal volume DMSO was used as solvent control. A: M210 vs XOM2_1 h; B: DMSO_2h vs OCA_2h; C: DMSO_4h vs OCA_4h; D: DMSO_6 h vs OCA_6 h; E: DMSO_8h vs OCA_8h. Values represent the levels of expression compared to that of bacterial cells cultured in M210 medium overnight (for which the level of expression was set equal to 1.00). Asterisks indicate statistically significant differences in the expression levels (Student’s t-test). * P < 0.05; ** P < 0.01. (TIF 10006 kb) [file 12866_2019_1532_MOESM1_ESM.tif]

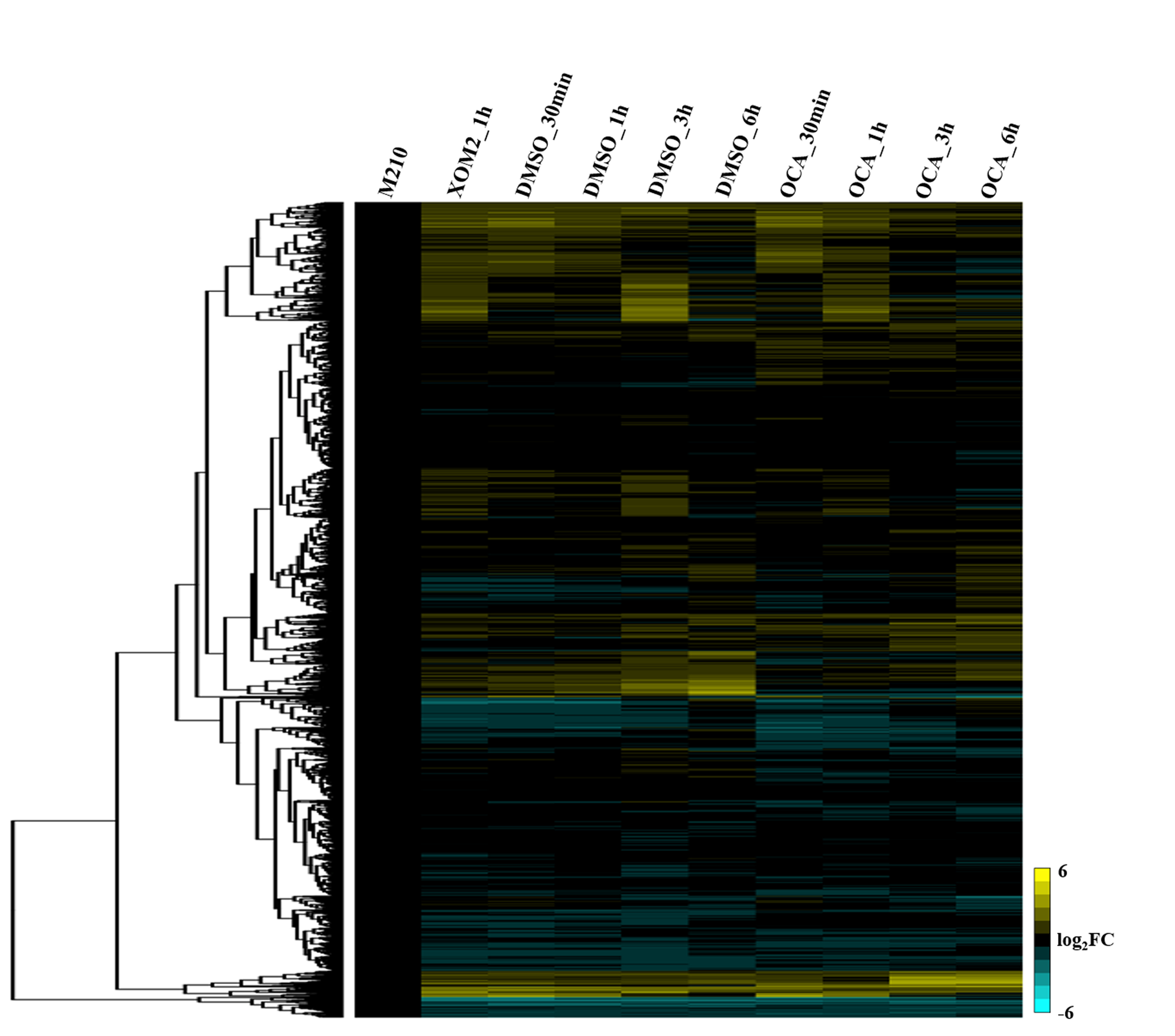

Supplement: Supplementary file 2 — Figure S2. Heatmap of OCA effects on Xoo PXO99A transcriptome. (TIF 10232 kb) [file 12866_2019_1532_MOESM2_ESM.tif]

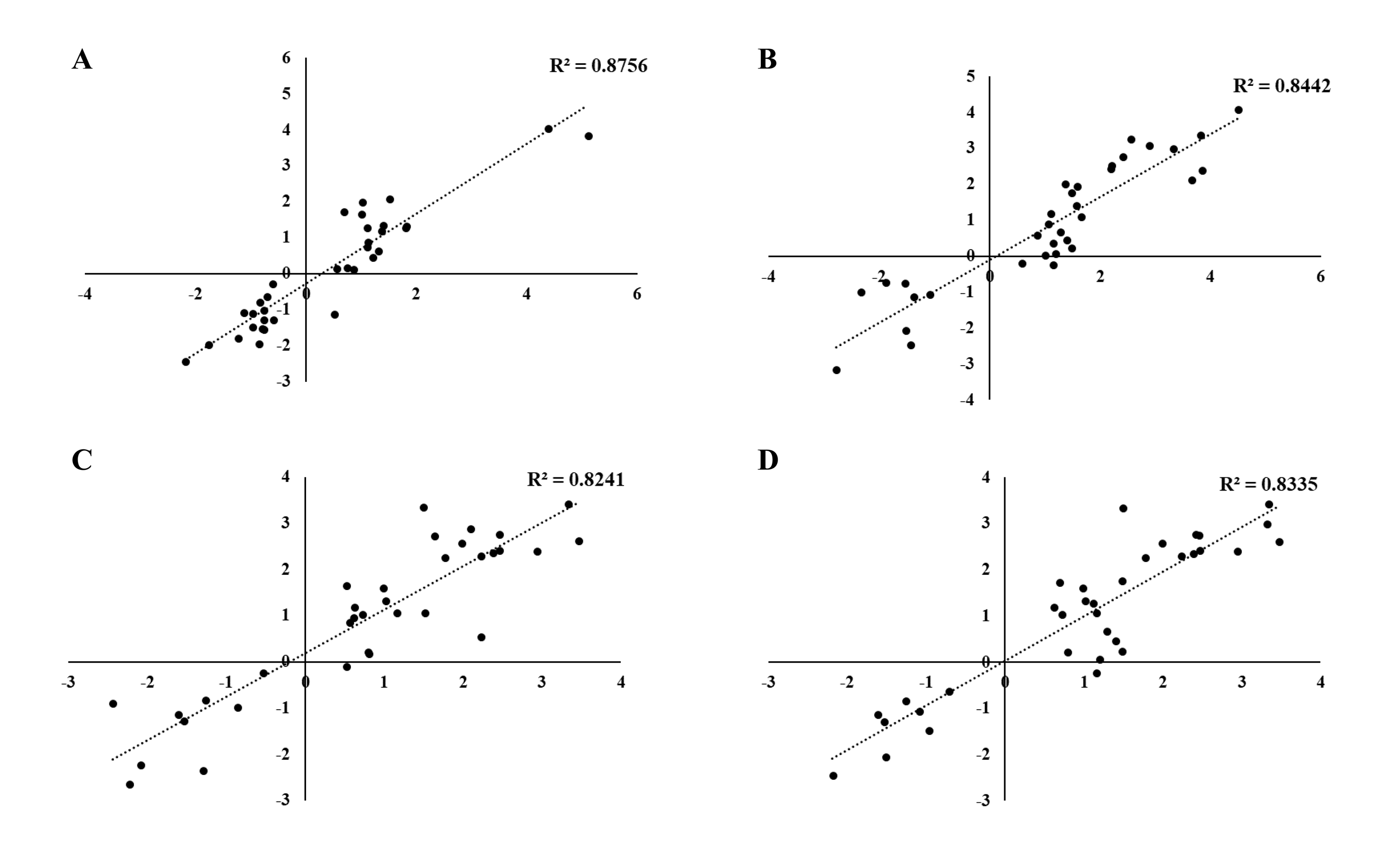

Supplement: Supplementary file 4 — Figure S3. Correlation analysis between qRT-PCR and RNA-seq data of 32 randomly selected genes. A, B, C and D represented 30 min, 1 h, 3 h and 6 h after OCA treatment, respectively. (TIF 215 kb) [file 12866_2019_1532_MOESM4_ESM.tif]

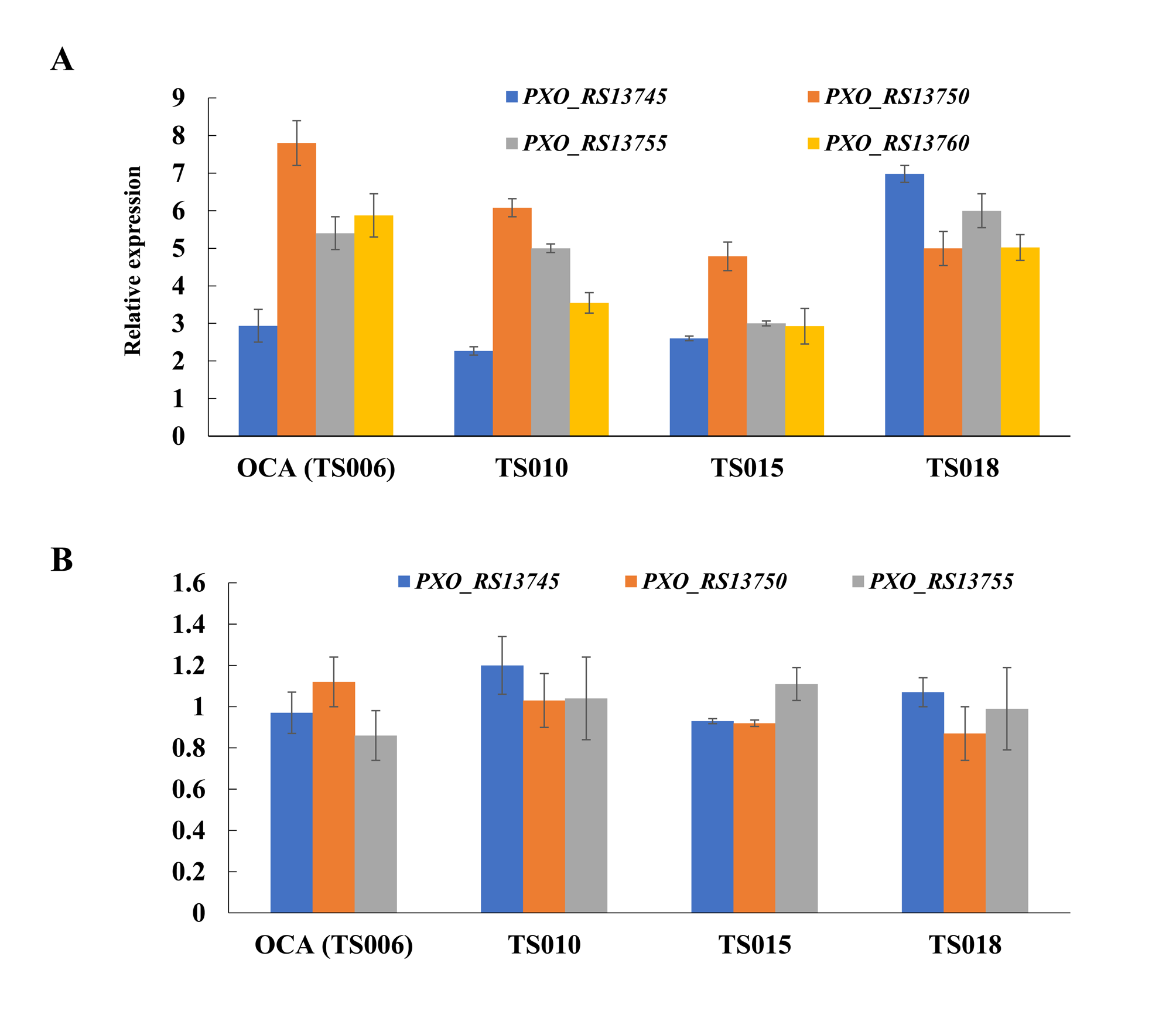

Supplement: Supplementary file 6 — Figure S4. Relative mRNA levels of the multidrug transporter gene cluster under treatment of different T3SS inhibitors were measured by qRT-PCR. (A) Relative mRNA levels of the multidrug transporter gene cluster in Xoo PXO99A incubated with four T3SS inhibitors respectively. (B) Relative mRNA levels of the multidrug transporter gene cluster in ΔPXO_RS13760 incubated with four T3SS inhibitors respectively. (TIF 10256 kb) [file 12866_2019_1532_MOESM6_ESM.tif]

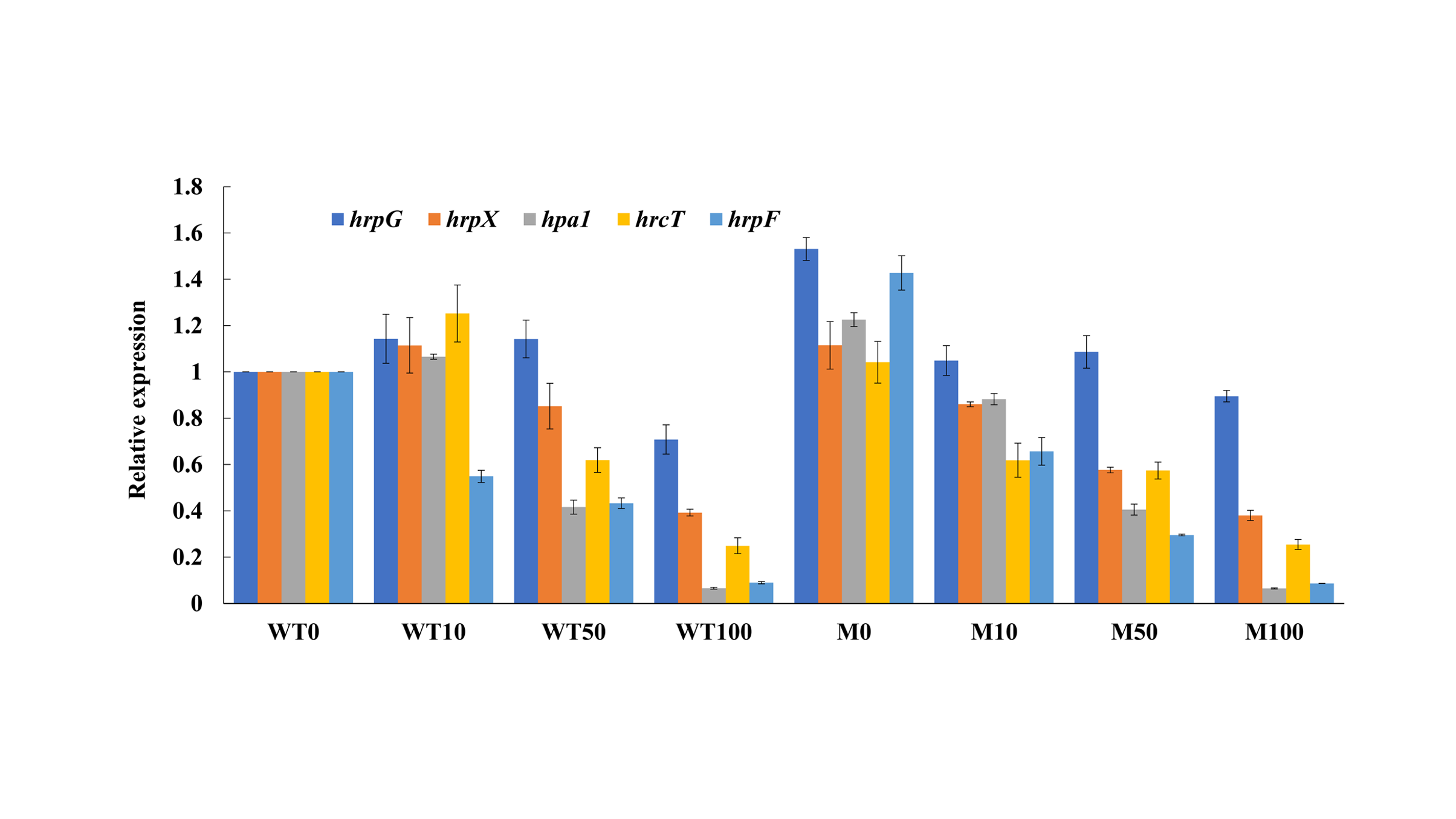

Supplement: Supplementary file 7 — Figure S5. Relative mRNA levels of hrp genes in Xoo incubated with OCA under different concentrations were measured by qRT-PCR. WT0, Xoo PXO99A wild type strain incubated without OCA; WT10, Xoo PXO99A wild type strain incubated with 10 μM OCA; WT50, Xoo PXO99A wild type strain incubated with 50 μM OCA; WT100, Xoo PXO99A wild type strain incubated with 100 μM OCA; M0, PXO_RS13760 deletion mutant incubated without OCA; M10, PXO_RS13760 deletion mutant incubated with 10 μM OCA; M50, PXO_RS13760 deletion mutant incubated with 50 μM OCA; M100, PXO_RS13760 deletion mutant incubated with 100 μM OCA. (TIF 9587 kb) [file 12866_2019_1532_MOESM7_ESM.tif]
