# Supplementary material for: Transcriptional responses of Xanthomonas oryzae pv. oryzae to type III secretion system inhibitor ortho-coumaric acid
Source: BMC Microbiol. 2019 Jul 15;19:163. doi: 10.1186/s12866-019-1532-5 (PMC6631524; doi:10.1186/s12866-019-1532-5)
Supplement: Supplementary file 10 — Table S5. Primers used in this study. (DOCX 25 kb) [file 12866_2019_1532_MOESM10_ESM.docx]

Table S5 Primers used in this study

| Primer name | Sequences（5’-3’） | Description of amplified product |
| --- | --- | --- |

| *hrpG*-F | TGTCCACCTGATGAACGACCCT |  |
| --- | --- | --- |
| *hrpG*-R | GGCGAATGCCGCAACGAA | *hrpG* gene fragment |
| *hrpX*-F | AGGCACTGACCCACTTTC |  |
| *hrpX*-R | ATCGGAAGCACCACTCTC | *hrpX* gene fragment |
| *hpa1*-F | AAGCCAGGACACAACGTTCG |  |
| *hpa1*-R | GAAGCAGGGCCGAGATGAG | *hpa1* gene fragment |
| *hrcC*-F | GCGCTTTCCGGTGCGTTAC |  |
| *hrcC*-R | CCCTGCTCGACCTGCTTGG | *hrcC* gene fragment |
| *hrcT*-F | AGGGCGTGTCGCTGTTGACC |  |
| *hrcT*-R | CGCGATTCCGGGAAGACTGT | *hrcT* gene fragment |
| *hrpF*-F | AGTCCGGCGTGCTCATCG |  |
| *hrpF*-R | AGTGCCCACCGCAGTTGA | *hrpF* gene fragment |

| PXO_RS00195-F | CACGCCTTGAACGATGAGG | |  |
| --- | --- | --- | --- |
| PXO_RS00195-R | ACGCTGCTGGGTTTGCTT | *PXO_RS00195* gene fragment | |
| PXO_RS00575-F  PXO_RS00575-R | CTGCGGAGCGAACTGGATG  AACACGCGGAAACCCTTGC | *PXO_RS00575* gene fragment | |
| PXO_RS01065-F | ATTCGCTCAATGCAGCGTAGT |  | |
| PXO_RS01065-R | CTTCCGTCGCCATAGTCCAG | *PXO_RS01065* gene fragment | |
| PXO_RS01345-F | CGGCGTGTCGGGATTTCA |  | |
| PXO_RS01345-R | GGCGTACCGCTCGGATTG | *PXO_RS01345* gene fragment | |
| PXO_RS01530-F | GGCATTTTCTCCAAAGTCCACG |  | |
| PXO_RS01530-R | CGGGTTGCCCAGAAATCGA | *PXO_RS01530* gene fragment | |
| PXO_RS02265-F | CGTCTGGGCTGGCTCAAT |  | |
| PXO_RS02265-R | CATCTCCATCACTTCACGCTCT | *PXO_RS02265* gene fragment | |
| PXO_RS02595-F | TTCTCGCTGATGTTCGCGTTCT |  | |
| PXO_RS02595-R | TCCGCCTGTGATGCCTTCC | *PXO_RS02595* gene fragment | |
| PXO_RS02690-F | CTGCCGATGCAAGCCAAGC |  | |
| PXO_RS02690-R | ACATCCACCTTCTGCGAGCC | *PXO_RS02690* gene fragment | |
| PXO_RS02955-F | AACGTAGTATTCGCCGACTGG |  | |
| PXO_RS02955-R | TACGCAGCGAGGAAAGCA | *PXO_RS02955* gene fragment | |
| PXO_RS03000-F | TTGCTGAGCGATGTAGGCG |  | |
| PXO_RS03000-R | GCTCGGGCATGTAGTAATACGG | *PXO_RS03000* gene fragment | |
| PXO_RS03810-F | CTGGCGGGTTACGGTTTG |  | |
| PXO_RS03810-R | GCATCTGGCTGTATTGGGTC | *PXO_RS03810* gene fragment | |

Table S2 (Continued)

| Primer name | Sequences（5’-3’） | Description of amplified product |
| --- | --- | --- |
| PXO_RS04370-F | TGCCAACGCGATGATGTG |  |
| PXO_RS04370-R | CAGCAATGCCGACCCAAT | *PXO_RS04370* gene fragment |
| PXO_RS06020-F | CCCAGTTGATGACCAATACGG |  |
| PXO_RS06020-R | AGCCAGCCCAGCAAGAAAA | *PXO_RS06020* gene fragment |
| PXO_RS06360-F | GGCAGCTTCGATGCGATTT |  |
| PXO_RS06360-R | CACCCGATTTGAGGTTGGA | *PXO_RS06360* gene fragment |
| PXO_RS06370-F | CCTCACGTTTCTGCTGGGTG |  |
| PXO_RS06370-R | TGGGGACGCGGTATTCGA | *PXO_RS06370* gene fragment |
| PXO_RS07440-F | TGATGCGGTTGGCGAAGT |  |
| PXO_RS07440-R | CAAGCATGGTGCGGAAGG | *PXO_RS07440* gene fragment |
| PXO_RS08045-F | GCTCAACGCTGAACTCGC |  |
| PXO_RS08045-R | TCGGCTTGCCATCCCATT | *PXO_RS08045* gene fragment |
| PXO_RS08365-F | AAGCTGCGCCTGAAAGAA |  |
| PXO_RS08365-R | GGAATCACGTCGGAAACG | *PXO_RS08365* gene fragment |
| PXO_RS08690-F | GACCCACTCCACCAAGAAGAA |  |
| PXO_RS08690-R | ACCGACCACGCCGACATA | *PXO_RS08690* gene fragment |
| PXO_RS08700-F | ATGATCGTCGGGCTGGATTT |  |
| PXO_RS08700-R | CGAACGCACGCAAGAAGG | *PXO_RS08700* gene fragment |
| PXO_RS08750-F | CCTGCCGTCACAGGTCTACAA |  |
| PXO_RS08750-R | GGGCGCACCAACAGATTG | *PXO_RS08750* gene fragment |
| PXO_RS09065-F | CGCCTGACGACGCACAAT |  |
| PXO_RS09065-R | GCACTCAGTGACTGCCAGGTTT | *PXO_RS09065* gene fragment |
| PXO_RS10415-F | AGAGCTGGCCTCGCTAGATG |  |
| PXO_RS10415-R | ATGCGGTTGGCGTTGATT | *PXO_RS10415* gene fragment |
| PXO_RS13745-F | GCTGCATCGCTCAACAACA |  |
| PXO_RS13745-R | GCCAGAAAGGTCCAACCAAG | *PXO_RS13745* gene fragment |
| PXO_RS13750-F | GCTGCGGATCGGGTTGTCT |  |
| PXO_RS13750-R | AGCGTTGGCGAGCTTGGTC | *PXO_RS13750* gene fragment |
| PXO_RS13760-F | TCAGGTACGTGATGCGCTAATG |  |
| PXO_RS13760-R | CGTAGTGGCTGAAGCCGAAAT | *PXO_RS13760* gene fragment |
| PXO_RS14960-F | ACGGTGAAAGCCTCGGTGAC |  |
| PXO_RS14960-R | CCACTTTGGCTGAGGTGTCGT | *PXO_RS14960* gene fragment |
| PXO_RS16275-F | GTGGTACAGCGCAATACAACATCA |  |
| PXO_RS16275-R | CTGCCTGGTTCAACGTCAGC | *PXO_RS16275* gene fragment |
| PXO_RS18090-F | TGGCTACTTCGCACAGCACA |  |
| PXO_RS18090-R | GCGGAACGCCTGATTGGAG | *PXO_RS18090* gene fragment |
| PXO_RS19145-F | GGTTGCACGGCTCATCGA |  |
| PXO_RS19145-R | TCACCACGGTTTCACCATCT | *PXO_RS19145* gene fragment |

Table S2 (Continued)

| Primer name | Sequences（5’-3’） | Description of amplified product |
| --- | --- | --- |
| PXO_RS19165-F | ATGCGCCGTTACGGACTC |  |
| PXO_RS19165-R | CCTCGACCTCGTAGCTTTCC | PXO_RS19165 gene fragment |
| PXO_RS20450-F | ACTGGGCTATGCAGGAGTCAG |  |
| PXO_RS20450-R | ACGGATCGCCTCGGAAAA | PXO_RS20450 gene fragment |
| gyrB-F | GGCGAGCACAATGGCATT |  |
| gyrB-R | CCATCCTTCTGCGGGATGT | gyrB gene fragment |
| PXO_RS13760US_F | CGGGATCCAGTCGGCCCATTGCTCCA |  |
| PXO_RS13760US_R | CGGAATTCATTCATGCACTGCATTTTTTCC | PXO_RS13760 upstream |
| PXO_RS13760DS_F | CGGAATTCGCATGAACGCTGCTAGTTCCC |  |
| PXO_RS13760DS_R | ACGCGTCGACTGGGATAGAAGCGGGTCTTGG | PXO_RS13760 downstream |
